# The burden of poisoning in children hospitalised at a tertiary-level hospital in South Africa

**DOI:** 10.3389/fpubh.2023.1279036

**Published:** 2023-10-20

**Authors:** Mahtaab Khan, Fatima Solomon, Alane Izu, Pepukai Bengura, Grace Okudo, Basetsana Maroane, Nilesh Lala, Ziyaad Dangor

**Affiliations:** ^1^Department of Paediatrics and Child Health, Faculty of Health Sciences, University of the Witwatersrand, Johannesburg, South Africa; ^2^South African Medical Research Council: Vaccines and Infectious Diseases Analytics Unit, University of the Witwatersrand, Johannesburg, South Africa

**Keywords:** paediatric, poisoning, pesticides, organic solvents, public health

## Abstract

**Introduction:**

Globally, childhood poisoning, accounts for a significant proportion of emergency department admissions. There is a paucity of data from low- and middle-income countries on poisoning in children.

**Objective:**

To describe the incidence, case fatality rate, and types of poisoning in children admitted to a tertiary-level hospital in Johannesburg, South Africa.

**Methods:**

This was a retrospective descriptive study of children hospitalised with poisoning from January 2016 to December 2021 at Chris Hani Baragwanath Academic Hospital. Children were identified from a discharge summary database using ICD-10 codes that describe poisoning. Trends in incidence of poison exposure were reported.

**Results:**

Of the 60,901 admissions during the study period, 2,652 (4.4%) children were diagnosed with poisoning. Most (71.3%) children were less than 5 years of age and 55% were male. The incidence of poisoning per 100,000 was highest at 108.4 (95% CI: 104.3–112.6) in 2019 and decreased to 77.3 (95% CI: 73.9–80.7) in 2020 and 59.6 (95% CI: 56.3–62.5) in 2021. Main causes of poisoning were organic solvents (37.6%), medications (32.9%), and pesticides (17.5%). The overall case fatality rate was 2.1%. In a multivariate analysis, poisoning secondary to pesticides (aOR: 13.9; 95% CI: 4.52–60.8; *p* < 0.001), and unspecified agents (aOR: 12.7; 95% CI: 3.27–62.8; *p* < 0.001) were associated with an increased odds of death.

**Conclusion:**

We report a high prevalence of poisoning in children hospitalised in this tertiary-level hospital in South Africa. Public health measures to reduce the burden of organic solvents, medications and pesticide poisoning are urgently warranted.

## Introduction

Childhood injuries represent an immediate and growing global concern (1). Poisoning is among the leading causes of childhood injury globally, and a common reason to visit the emergency department (2, 3). A World Health Organisation (WHO) world report on childhood injury prevention noted that poisoning accounts for 10% of unintentional accidents in children less that 15-years-of-age (4).

The types of substances and pattern of poisoning vary across regions with a disproportionately four-fold higher mortality in children from low- and middle-income countries (LMICs) (4). Common substances include medications, either prescription or over the counter, pesticides, recreational drugs, or household cleaning products. In LMICs, accidental hydrocarbon (such as paraffin/kerosene for lighting and fuel) ingestion is a common reason for seeking emergency care (5). Pesticide ingestion or exposure occurs more frequently in countries where agriculture is a source of income. Household pesticides are often used in LMICs with inadequate sanitation and overcrowding. Furthermore, people living in LMICs have an increase availability of non-registered cheaper pesticides from commercial markets (6).

In South Africa, several studies undertaken in multiple areas across the country have shown that poisoning from hydrocarbons, kerosene or paraffin is still the most common agent, but there is a rise in poisoning secondary to pesticides (6–8).

The restrictive measures and emotional stressors of the COVID-19 pandemic may have influenced the patterns of poisoning. In Canada, despite the significant reduction in paediatric emergency department visits, the number of visits for poisoning doubled during the pandemic (9). Similarly, data from Poison Information Helplines showed an increased number of calls from the public regarding poisoning, most of these were reported to be accidental (10).

We therefore described the burden (before COVID-19 and during the pandemic) and types of poisoning in children hospitalised at a tertiary-level academic hospital in a low-resourced community in South Africa.

## Methods

A retrospective, descriptive study was undertaken at the Chris Hani Baragwanath Academic Hospital (CHBAH) from January 2016 to December 2021. CHBAH is situated south of Johannesburg, in the peri-urban suburb of Soweto, and is a government-funded hospital which provides care to the community of Soweto and its surrounding areas. Children with a history of exposure to a poison, or clinical signs of poisoning are transferred from primary health care clinics or a district hospital to CHBAH for assessment and management. On presentation, these children are assessed by the attending clinician, and most are hospitalised for stabilisation or observation depending on the severity of the presentation. Clinicians have access to an online Afritox Poison Information Database (courtesy of the University of Cape Town) to identify toxidromes and the management thereof.

South Africa is an upper middle-income country with a high GINI co-efficient (11). There is a high rate of unemployment and approximately 29% of the population of Soweto live under $2 per day (12). Paraffin/ kerosene is often used for cooking and light, and there are no set regulations on the labelling of paraffin as a hazardous substance - these are often placed in non-labelled or freely available cooldrink or milk cartons in households. Furthermore, registered, and off-label pesticides are easily accessible to curb the rat/mice infestation in the informal settlements, and where garbage has been dumped in open areas.

Children less than 14 years-of-age admitted to the paediatric wards at CHBAH with poisoning were identified from an electronic database (managed by the Vaccines and Infectious Diseases Analytics Research Unit) using the international classification of diseases tenth revision (ICD 10) codes describing a poison exposure (Supplementary Table 1). Clinical and demographic characteristics were extracted from the database.

### Statistical analysis

For the analysis, the type of poison was classified into six groups: alcohol, medication, organic solvents (including paraffin), pesticides, other ingested agents, and unspecified toxins. We further stratified children into two age groups: children less than 5 years and 5 years – <14 years-of-age.

Trends in the incidence of poison exposure were calculated and expressed as the number of cases divided by the mid-year population estimates for region D (Soweto) and G (south of Soweto) of the Johannesburg metropolitan. We compared the incidence before COVID-19 (January 2016 to December 2019) to the incidence during the COVID-19 pandemic (January 2020 to December 2021).

Categorical variables were expressed as proportions or frequencies and compared using either the Chi-squared or Fisher’s exact tests. Univariable summaries (means, medians) were used to report continuous variables. A regression analysis was undertaken to determine the predictors of mortality and effect of the COVID-19 pandemic on poisoning. All variables from the univariate analysis were included in the multivariate analysis. Adjusted odds ratios and 95% confidence intervals were reported for. Data were analysed using STATA version 17.0. A value of *p* <0.05 was considered statistically significant. The study was approved by the University of Witwatersrand Human Research Ethics Committee (HREC number: M220744).

## Results

**Figure 1:**
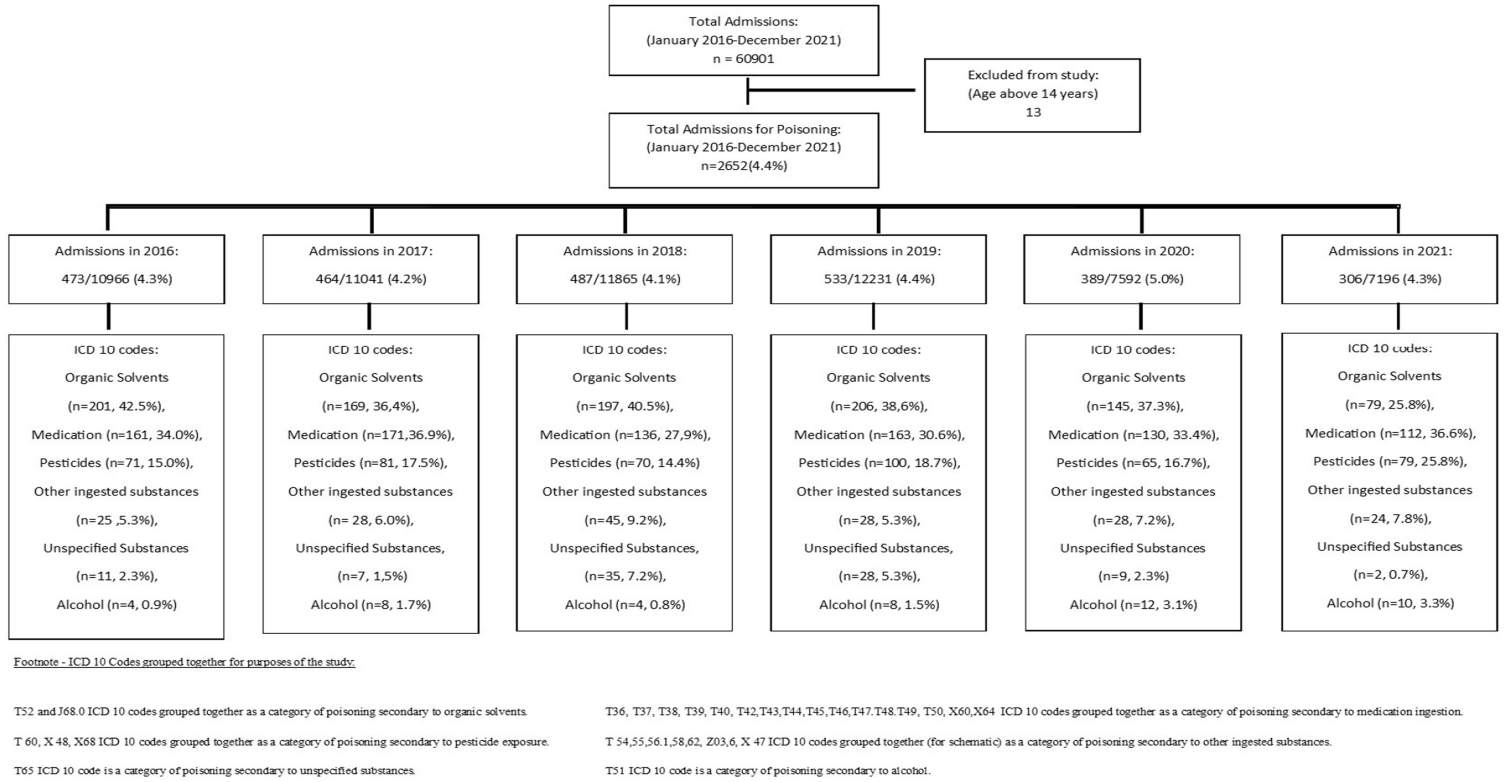
Flow diagram representing children admitted with poison exposure from January 2016 to December 2021.

**Figure 2:**
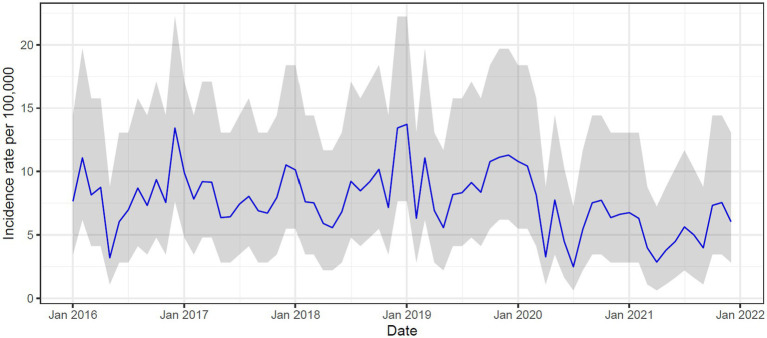
Monthly incidence rate of poison admissions from January 2016 to December 2021.

Of the 60,901 admissions to the paediatric wards during the study period, 2,652 admissions (4.4%) had a discharge diagnosis of poisoning (Figure 1). The most common type of poison was organic solvents (37.6%), followed by medications (32.9%) and pesticides (17.5%; Supplementary Table 1). The median age of admission was 2.8 years (±2.3 years), and 1,459 (55%) were male. Most (78.2%) cases occurred in children 1-5 years of age. The primary diagnosis was poisoning in the majority (93.9%) of cases (Table 1). For cases with a secondary diagnosis (6.1%) of poisoning, the primary diagnosis was captured most frequently as lower respiratory illness (30.7%), acute gastroenteritis (22.1%), seizures (16.0%), and septicaemia (6.8%). Ninety-three (3.5%) children required invasive ventilation; of these 54 (59.1% of ventilated patients) were transferred to an ICU and the remainder were in the high care area. Of the 39 children that remained in the high care, there was no available ICU bed in 22 (56.4%), 8 (20.5%) were assessed as poor candidates for ICU, 5 (12.8%) had no reason stated, and 4 (10.3%) were extubated and did not require ICU. Children with pesticide poisoning were 10-fold (95% CI: 6.52–15.75) more likely to require high care or ICU admission. The main indications for ventilation were central nervous system (45.0%) and respiratory signs (36.0%; Figure 2).

**Table 1:** Clinical characteristics of children with poisoning, stratified by poison type.

Variables	Overall	Organic solvents	Medications	Pesticides	Other	Unspecified	Alcohol
	*n* = 2,652	997 (37.6%)	873 (32.9%)	466 (17.6%)	178 (6.7%)	92 (3.5%)	46 (1.7%)
Gender							
Male	1,459 (55.0%)	606 (60.8%)	444 (50.9%)	240 (51.5%)	93 (54.4%)	50 (54.3%)	26 (56.5%)
Female	1,177 (44.4%)	384 (38.5%)	426 (48.8%)	223 (47.9%)	84 (47.2%)	42 (45.7%)	18 (39.1%)
Unknown	16 (0.6%)	7 (0.7%)	3 (0.3%)	3 (0.6%)	1 (0.6%)	0	2 (4.4%)
Age							
< 5 years	2073 (78.2%)	943 (94.6%)	589 (67.5%)	333 (71.5%)	115 (64.6%)	74 (80.3%)	19 (41.3%)
5 years - < 14 years	579 (21.8%)	54 (5.4%)	284 (32.5%)	133 (28.5%)	63 (35.4%)	18 (19.6%)	27 (59.7%)
Diagnosis							
Primary diagnosis of poisoning	2,489 (93.9%)	978 (98.1%)	791 (90.6%)	451 (96.8%)	158 (88.8%)	68 (73.9%)	43 (97.5%)
Secondary diagnosis of poisoning	163 (6.1%)	19 (1.9%)	82 (9.4%)	15 (3.2%)	20 (11.2%)	24 (26.1%)	3 (6.5%)
Required High Care or ICU	93(3.5%)	14(1.4%)	14(1.6%)	61(13.1%)	3(1.7%)	1(1.1%)	0
Median (IQR) Length of Stay (Days)	1 (1–2)	1(1–1)	1(1–3)	1(1–4)	1(1–2)	1(1–3)	1 (1–2)
Outcome							
Discharged	2,574 (97.1%)	987 (99.0%)	851 (97.5%)	436 (93.6%)	172 (96.6%)	83 (90.2%)	46 (100%)
Demised	56 (2.1%)	4 (0.4%)	15 (1.7%)	26 (5.6%)	2 (1.1%)	9 (9.8%)	0

**Figure 3:**
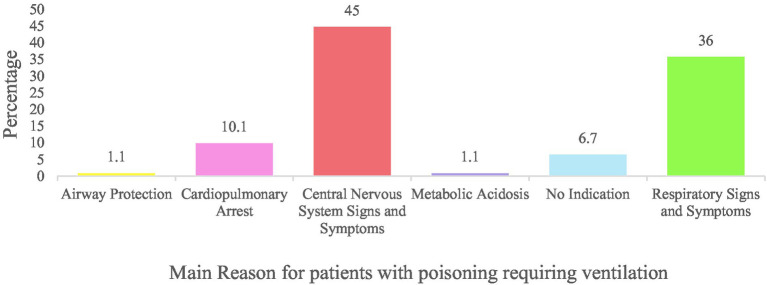
Main reasons for patients with poisoning requiring ventilation.

The incidence of poisoning per 100,000 was highest at 108.4 (95% CI: 104.3–112.6) in 2019 and decreased to 77.3 (95% CI: 73.9–80.7) in 2020 and 59.6 (95%CI: 56.6.0–62.5) in 2021 (Supplementary Figure 1). In the pre-covid years (2016–2019), peaks in incidence were noted in December and January, but this was less pronounced in 2020 and 2021 (Figure 3).

The overall case fatality rate for the study was 2.1%. Children with poisoning secondary to unspecified agents had the highest fatality rate (9.8%), followed by poisoning secondary to pesticides (5.6%) and medications (1.7%). In a multivariate analysis, children admitted to high care or ICU were more likely to demise compared to those who were admitted to the general paediatric wards (aOR: 15.8; 95%CI: 6.12–41.70; *p* < 0.001; Table 2) Poisoning secondary to pesticides (aOR: 13.9: 95% CI: 4.52--60.8; *p* < 0.001), and unspecified agents (aOR: 12.7, 95% CI: 3.27–62.8; p < 0.001) had an increased odds of death compared to the reference (Table 2).

**Table 2:** Predictors of mortality in children with poisoning.

Multivariable logistic regression (Demised vs. Discharged vs.)			
Characteristic	aOR^1^	95% CI^2^	Value of *p*
Poison agent			
Organic solvents	Ref	Ref	
Medication	3.48	1.08–15.5	0.057
Pesticides	13.9	4.52–60.8	<0.001
Other ingested substances	1.26	0.14–8.92	0.800
Unspecified	12.7	3.27–62.8	<0.001
Age			
<= 5 years	Ref	Ref	
> 5 years	1.47	0.75–2.79	0.300
Gender			
Male	Ref	Ref	
Female	0.78	0.42–1.41	0.400
Diagnosis			
Secondary	Ref	Ref	
Primary	0.05	0.03–0.11	<0.001
Length of stay			
<= 3 days	Ref	Ref	
> 3 days	0.17	0.06–0.40	<0.001
HCA admission			
No	Ref	Ref	
Yes	15.8	6.12–41.7	<0.001

aOR^1^, Adjusted Odds Ratio; CI^2^, Confidence Interval.

The prevalence of poisoning increased to 5.0% in 2020, albeit not significant, and returned to pre-pandemic rates in 2021. For the years 2020 and 2021, medication ingestion (33.4 and 36.6%, respectively) was among the main cause of poisoning, followed by organic solvents and pesticides. There was an increased odds ratio of children being hospitalised with alcohol (aOR: 3.23 95% CI: 1.72–6.09; *p* < 0.001). and pesticide poisoning (aOR: 1.40 95% CI: 1.07–1.81; *p* < 0.013) in the COVID-19 pandemic (2020–2021) compared to the pre COVID-19 years (Table 3). The mortality rate during the COVID-19 pandemic also increased compared to the pre COVID-19 years (2.6% vs. 1.9%; *p* = 0.001).

**Table 3:** Factors associated with poisoning in children during the COVID-19 pandemic.

Multivariable logistic regression (COVID vs. Non-COVID years)			
Characteristic	aOR^1^	95% CI^2^	Value of *p*
Poison agent			
Organic solvents	Ref	Ref	
Alcohol	3.23	1.72–6.09	<0.001
Medication	1.24	0.99–1.54	0.063
Pesticides	1.40	1.07–1.81	0.013
Other ingested substances	1.36	0.94–1.96	0.100
Unspecified	0.47	0.23–0.86	0.022
Age			
<= 5 years	Ref	Ref	
> 5 years	1.17	0.93–1.45	0.200
Gender			
Male	Ref	Ref	
Female	1.03	0.86–1.23	0.700
Diagnosis			
Secondary	Ref	Ref	
Primary	1.22	0.83–1.83	0.300
Length of stay			
<= 3 days	Ref	Ref	
> 3 days	1.35	1.03–1.75	0.026
HCA admission			
No	Ref	Ref	
Yes	1.09	0.66–1.77	0.7

aOR^1^, Adjusted Odds Ratio, CI^2^, Confidence Interval.

## Discussion

The study showed that one in every 25 children hospitalised at a large tertiary-level hospital in South Africa are poisoned, a third from organic solvents (such as paraffin) and medications, respectively, and alarmingly a further 18% from pesticides. Furthermore, 56 (2.1%) children demised from poisoning which is four-fold higher than the 0.5% reported in high-income countries (13). The mortality rate for children with pesticide poisoning was also more than double in the study. These findings call for stricter public health interventions to be implemented in LMIC settings. In contrast to other studies (9), our study showed a lower incidence of poisoning during the COVID-19 pandemic that may in part be attributed to better supervision of young children by caregivers present in the home environment during the national lockdown periods.

The prevalence of hospitalised children with poisoning in our setting was approximately 4.4% which is higher than a study conducted in France (3% prevalence) (14). Most studies differ in the reporting and surveillance systems to determine incidence rates (15). The annual incidence of poisoning in the US was reported higher (168 per 100,000 non-fatal paediatric poisoning) than our study (16), which may be from better reporting or more children seeking health care for poisoning compared to South Africa.

In high-income countries, pesticide poisoning was reported in 7.8% of children less than 15 years of age. There is a paucity of data from Africa; however, a study from 2016 showed that pesticide poisoning was present in 23.2% of South African children under the age of 10 (17). In our study, pesticides, including organophosphates were a common (18%) cause of poisoning. Ingestion of pesticides may occur accidentally in young children who have an increased hand to mouth activity and exploring their surroundings – this is particularly relevant in our setting where these pesticides are used to curb rodent infestation (6, 13). Pesticides could also be used for intentional poisoning, particularly among adolescents (18). In our study, we were unable to determine the intent of poisoning but saw an increase of pesticide poisoning from 17% pre-covid to 31% during the COVID-19 pandemic, and a shift of overall poisoning towards the older age groups. This is in keeping with studies that reported an increase in suicide rates among adolescents and adults during the pandemic that were largely attributable to school failures, death of family members, loneliness, and a diagnosis of COVID-19 (19, 20).

Organic solvent poisoning, including paraffin, is a major public health concern in South Africa (21). Paraffin ingestion was also a leading cause (20%) of poisoning at the Red Cross War Memorial Hospital in Cape Town between 2003 and 2015 (22). Paraffin ingestion can lead to chemical pneumonitis, but severe disease and mortality rates are low (23). Our study is similar to previous studies in which children with paraffin poisoning have a short hospital stay (median: 1 day; IQR:1–4) and low mortality rate of 0.2%. Younger children are more likely to accidentally ingest paraffin from their inability to recognise it as unsafe (13). Therefore, educational programmes in LMICs need to focus on labelling of paraffin, child proof lids and storage in safe places that are inaccessible to young children.

Ingestion of medication either via prescription or over the counter was also noted to be common in our setting. In the US, half of all poison centre calls are from exposure to pharmaceutical agents, and 95% of these exposures are accidental, and in children less than 5-years-of-age (16). Similarly, we found that most medication poisoning was in children less than 5-years-of-age. Poisoning secondary to diuretics and other ingested agents including unspecified drugs, medicaments and biological substances accounted for 47% of poisoning secondary to medication, followed by anti-epileptics, sedatives, and other hypnotics. Analgesic medication, which has been noted to be a common cause of poisoning globally, was less common in our setting (3). Furthermore, we found a high mortality in patients less than 5 years-of-age presenting with medication ingestion, particularly in children less than 1 year old (Supplementary Figure 3). This higher mortality rate could be due to the difference in physiological mechanisms in younger children including the organs and metabolic systems still developing resulting in a delay in elimination of the toxin and the blood brain barrier being less mature, allowing substances to penetrate more easily.

This study had some limitations. Using ICD 10 codes for analysis did not allow us to accurately ascertain whether the poisonings were intentional or unintentional. As this was a retrospective review, important variables such as time of arrival post poison exposure, circumstances relating to exposure and clinical manifestations of the exposure could not be explored.

In conclusion, we report a high prevalence of poisoning and death in children hospitalised in this tertiary-level hospital in South Africa. Organic solvents and pesticide poisoning are commonly encountered in South Africa due to the socio-economic disposition of the country. Public health measures are required to decrease the burden of poisoning in children.

## Data availability statement

The raw data supporting the conclusions of this article will be made available by the authors, without undue reservation.

## Ethics statement

The studies involving humans were approved by University of Witwatersrand Ethics Committee. The studies were conducted in accordance with the local legislation and institutional requirements. Written informed consent for participation was not required from the participants or the participants' legal guardians/next of kin in accordance with the national legislation and institutional requirements.

## Author contributions

MK: Conceptualization, Data curation, Formal analysis, Investigation, Methodology, Resources, Writing – original draft, Writing – review & editing. FS: Data curation, Writing – review & editing. AI: Data curation, Formal analysis, Writing – review & editing. PB: Formal analysis, Methodology, Writing – review & editing. GO: Data curation, Writing – review & editing. BM: Data curation, Writing – review & editing. NL: Conceptualization, Data curation, Formal analysis, Investigation, Methodology, Resources, Supervision, Writing – original draft, Writing – review & editing. ZD: Conceptualization, Data curation, Formal analysis, Investigation, Methodology, Resources, Supervision, Writing – original draft, Writing – review & editing.
