# Supplementary figures and images for: The burden of poisoning in children hospitalised at a tertiary-level hospital in South Africa
Source: Front Public Health. 2023 Oct 20;11:1279036. doi: 10.3389/fpubh.2023.1279036 (PMC10623415; doi:10.3389/fpubh.2023.1279036)

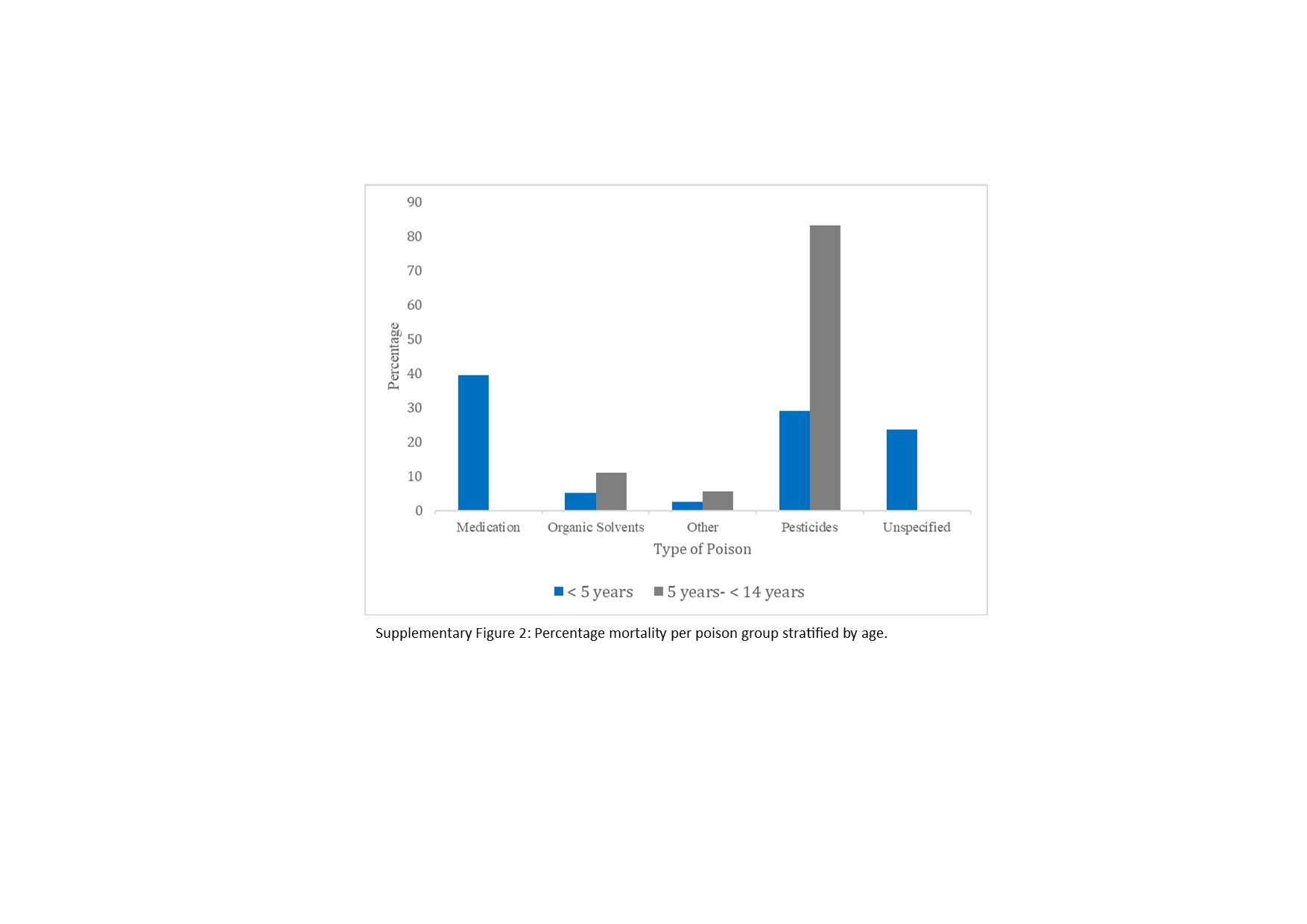

Supplement: Supplementary file 1 [file Image_1.JPEG]

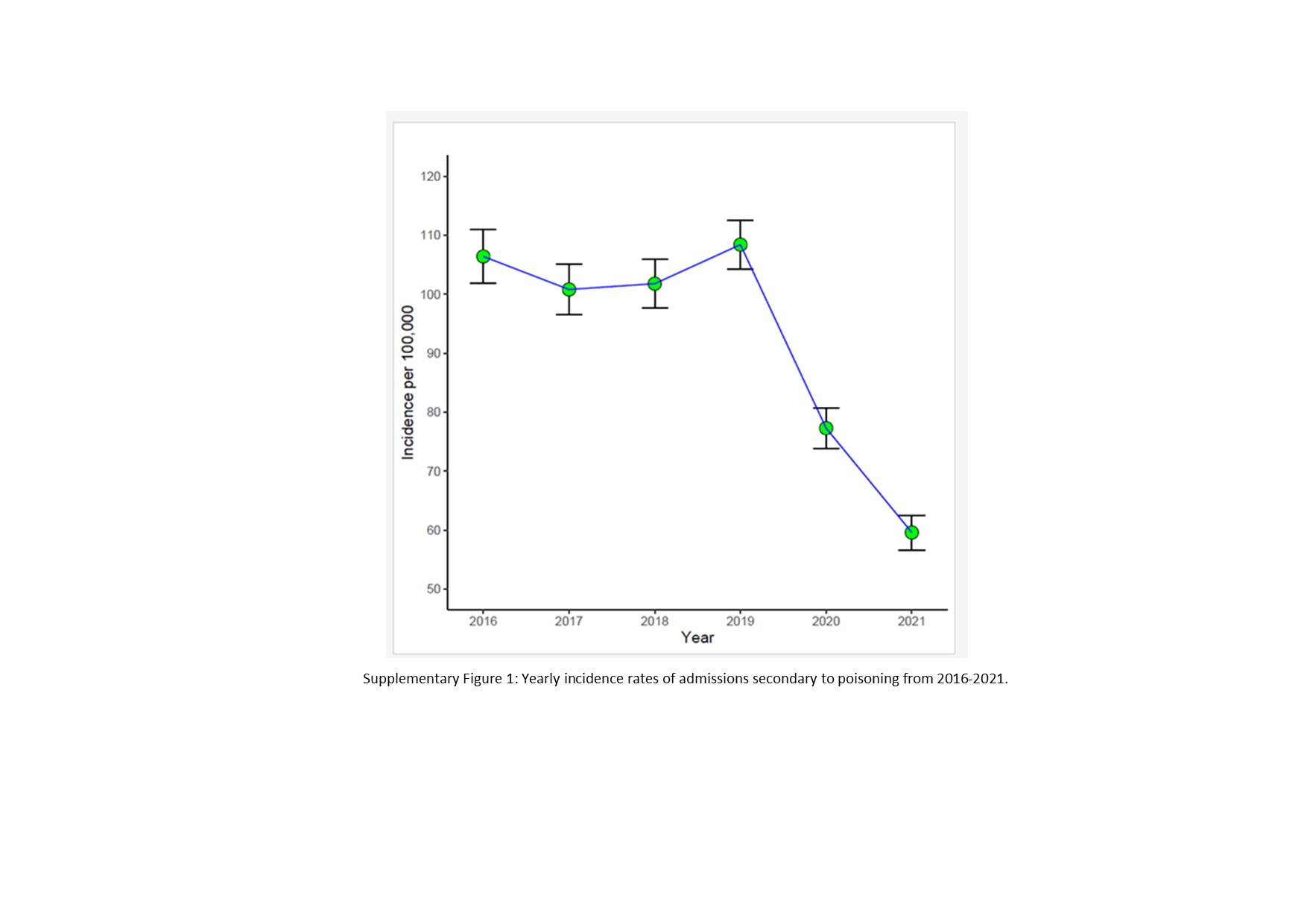

Supplement: Supplementary file 2 [file Image_2.JPEG]
