# Supplementary material for: The burden of poisoning in children hospitalised at a tertiary-level hospital in South Africa
Source: Front Public Health. 2023 Oct 20;11:1279036. doi: 10.3389/fpubh.2023.1279036 (PMC10623415; doi:10.3389/fpubh.2023.1279036)
Supplement: Supplementary file 3 [file Table_1.pdf]

Supplementary Table 1 : ICD-10 coding for poison admissions by year 2016 -2021

Description	2016	2017	2018	2019	2020	2021	Total (%)
**POISONING SECONDARY TO ORGANIC SOLVENT EXPOSURE** (ICD 10 codes J68.0 & T52)	**201 (42.5%)**	**169 (36.4%)**	**197 (40.5%)**	**206 (38.6%)**	**145 (37.3%)**	**79(25.8%)**	**997(37.6%)**
**POISONING SECONDARY TO MEDICATION EXPOSURE** (ICD 10 codes T36, T37, T38, T39, T40, T42, T44, T45, T46, T47, T48, T49, T50, X60, X64)	**161 (34.1%)**	**171 (36.9%)**	**136 (27.9%)**	**163 (30.5%)**	**130 (33.4%)**	**112 (36.6%)**	**873(32.9%)**
Poisoning by diuretics and other ingested agents unspecified drugs, medicaments and biological substances.	84(17.8%)	79(17.0%)	66(13.6%)	80(15.0%)	58(14.9%)	43(14.0%)	410 (15.5)
Poisoning by anti-epileptic, sedative- hypnotic and anti-parkinsonism drugs	24(5.1%)	38(8.2%)	20(4.1%)	20(3.7%)	27(6.9%)	13(4.3%)	142(5.4)
Poisoning by psychotropic drugs, not elsewhere classified	15(3.2%)	10(2.2%)	16(3.3%)	22(4.1%)	14(3.6%)	16(5.2%)	93(3.5)
Poisoning by nonopioid analgesics, antipyretics and antirheumatics	14(3.0%)	11(2.4%)	9(1.9%)	8(1.5%)	7(1.8%)	8(2.6%)	57 (2.2)
Poisoning by primarily systemic and haematological agents, not elsewhere classified	2(0.4%)	12(2.6%)	6(1.2%)	6(1.0%)	5(1.3%)	14(4.6%)	45(1.7)
Poisoning by systemic antibiotics, by other systemic anti-infectives.	3(0.6%)	5(1.1%)	7(1.4%)	8(1.5%)	4(1.0%)	4(1.3%)	31(1.2)
Poisoning by agents primarily affecting the cardiovascular system	4(0.8%)	6(1.3%)	4(0.8%)	6(1.0%)	6(1.5%)	3(1%)	29(1.1)
Poisoning by hormones and their synthetic substitutes and antagonists, not elsewhere classified	3(0.6%)	2(0.4%)	5(1.0%)	1(0.2%)	3(0.8%)	5(1.6%)	19 (0.7)
Poisoning by agents primarily acting on smooth and skeletal muscles and the respiratory system	5(1.1%)	2(0.4%)	1(0.2%)	4(0.8%)	0	2(0.7%)	14(0.5)
Poisoning by agents primarily affecting the gastrointestinal system	5(1.1%)	3(0.7%)	0	2(0.4%)	0	1(0.3%)	11(0.4)
Poisoning by narcotics and psychodysleptics (hallucinogens)	0	2(0.4%)	1(0.2%)	2(0.4%)	4(1.0%)	2(0.7%)	11(0.4)
Poisoning by systemic Antibiotics	1(0.2%)	0	1(0.2%)	1(0.2%)	0	0	3(0.1)
Poisoning by topical agents primarily affecting skin and mucous membranes and by ophthalmological, otorhinolaryngological and dental drugs	1(0.2%)	0	0	0	1(0.3%)	0	2 (0.1)
Poisoning by drugs primarily affecting the autonomic nervous system	0	0	0	1(0.2%)	0	1(0.3%)	2(0.1)
Intentional self-poisoning by exposure to other and unspecified drugs, medicaments and biological substances	0	0	0	2(0.4%)	0	0	2(0.1)
Accidental poisoning by and exposure to narcotics and psychodysleptics (hallucinogens), not elsewhere classified	0	0	0	0	1(0.3%)	0	1(0.1)
Intentional self-poisoning by and exposure to nonopioid analgesics, antipyretics and antirheumatics	0	1(0.2%)	0	0	0	0	1(0.1)
**POISONING SECONDARY TO PESTICIDE EXPOSURE** (ICD codes T60, X48, X68)	**71(15.0%)**	**81(17.5%)**	**70(14.4%)**	**100 (18.8%)**	**65(16.7%)**	**79(25.8%)**	**466(17.5%)**
**POISONING SECONDARY TO OTHER INGESTED SUBSTANCES EXPOSURE** (ICD 10 codes T51, T54, T56.1, T58, Z03,6, X47):	**25(5.3%)**	**28(6.0%)**	**45(9.2%)**	**28(5.3%)**	**28(7.2%)**	**24(7.8%)**	**178(6.7%)**
Observation for suspected toxic effect from ingested substance	6(1.3%)	19(4.1%)	30(6.1%)	24(4.5%)	18(4.7%)	8(2.6%)	105 (4.0)
Toxic effect of soaps and detergents	18(3.8%)	8(1.7%)	10(2.1%)	3(0.6%)	4(1.0%)	3(1%)	46 (1.7)
Toxic effect of carbon monoxide	0	0	2(0.4%)	0	6(1.5%)	7(2.2%)	15 (0.6)
Toxic effect of corrosive substances	1(0.2%)	0	2(0.4%)	0	0	1(0.3%)	4 (0.2)
Toxic effect of mercury and its compounds	0	0	0	0	0	3(1%)	3 (0.1)
Accidental poisoning by and exposure to other gases and vapors	0	1(0.2%)	0	0	0	2(0.7%)	3 (0.1)
Toxic effects of other noxious substances eaten as food	0	0	1(0.2%)	1(0.2%)	0	0	2(0.1)
**POISONING SECONDARY TO UNSPECIFIED SUBSTANCES EXPOSURE** (ICD 10 code T65)	**11(2.3%)**	**7(1.5%)**	**35(7.2%)**	**28(5.3%)**	**9(2.3%)**	**2(0.7%)**	**92(3.5)**
**POISONING SECONDARY TO ALCOHOL EXPOSURE** (ICD 10 code T51)	**4(0.8%)**	**8(1.7%)**	**4(0.8%)**	**8(1.5%)**	**12(3.1%)**	**10(3.3%)**	**46 (1.7)**
